# Inflammatory blood parameters as prognostic factors for implant-associated infection after primary total hip or knee arthroplasty: a systematic review

**DOI:** 10.1186/s12891-023-06500-z

**Published:** 2023-05-15

**Authors:** Petr Domecky, Anna Rejman Patkova, Katerina Mala-Ladova, Josef Maly

**Affiliations:** Department of Social and Clinical Pharmacy, Faculty of Pharmacy in Hradec Kralove, Charles University, Ak. Heyrovskeho 1203/8, 500 05 Hradec Kralove, Czech Republic

**Keywords:** Hip, Knee, Arthroplasty, Infection, Inflammatory blood parameters, Prognostic factor

## Abstract

**Background:**

Implant-associated infection (IAI) is a potential complication following total hip (THA) or knee arthroplasty (TKA). The initial phase of the inflammatory process can be measured by applying one of the inflammatory blood parameters (IBP). This systematic review aims to assess the response of IBP to trauma caused by orthopedic surgery and evaluate the clinical utility of quantitative measurements of IBP as prognostic factors for infection.

**Methods:**

All studies indexed in Ovid MEDLINE (PubMed), Ovid EMBASE, the Cochrane Library and the ISI Web of Science databases, from inception until January 31, 2020, were analyzed. Studies included were those on adults who underwent THA or TKA with minimum follow up of 30 days after surgery. In addition to minimum follow up, data on the prognostic factors for pre- or post-THA/TKA IAI were mandatory. The Quality Assessment of Diagnostic Accuracy tool (version 2) (QUADAS-2) and Standards for Reporting of Diagnostic Accuracy Studies guideline 2015 (STARD) were used for quality assessment.

**Results:**

Twelve studies fulfilled the inclusion and exclusion criteria. C-reactive protein was analyzed in seven studies, interleukin-6 in two studies and erythrocyte sedimentation rate in eight studies. White blood cell count and procalcitonin were analyzed in the only study. The overall quality of included studies was low. A potential for other cytokines (IL-1ra, IL-8) or MCP-1 was observed.

**Conclusions:**

This is the first systematic review of IBP response to orthopedic surgery which identified some IBP for pre/post-operative screening, despite insufficient data supporting their prognostic potential for patient risk stratification.

## Introduction

Although joint replacement is a successful treatment for osteoarthritis, there is some risk of potential complications, namely implant-associated infection (IAI) [1–4].

The IAI occurrence may be effectively reduced by various preoperative and postoperative measures [5, 6]. Therefore, these measures must be individualized for each patient by applying various predictive tools. Although predictive tools, such as the ACS (American College of Surgeons) surgical risk calculator or the NHSN (National Healthcare Safety Network) risk index are available, none of them estimates the risk of an early infection based on inflammatory blood parameters (IBP) [7, 8].

The beginning of the inflammatory process can be measured by applying one of the IBP, including interleukin-6 (IL-6), c-reactive protein (CRP), neutrophil-to-lymphocyte ratio (NLR), white blood cell count (WBC), erythrocyte sedimentation rate (ESR), and procalcitonin (PCT) [9–13].

The present systematic review aimed to identify studies that evaluated the IBP in patients with primary total hip arthroplasty (THA) and total knee arthroplasty (TKA). By analyzing the preoperative and postoperative values of IBP, clinicians would be able to identify patients at a higher risk for IAI and thus tailor their preoperative and postoperative measurement accordingly.

## Materials and methods

The systematic review was conducted following the PRISMA guidelines [14]. A protocol of this review was registered in the international prospective register for systematic reviews (PROSPERO, number: CRD42020147925) and published before the completion of this systematic review [15].

### Search strategy and study selection

All studies indexed in Ovid MEDLINE (PubMed), Ovid EMBASE, the Cochrane Library and ISI Web of Science databases from 1902 or inception until January 31, 2020, were analyzed. The search terms included both medical subject headings (MeSH) and keywords related to: IBP or risk factors, prosthesis-related infection or surgical site infection, and knee or hip arthroplasty. The complete search strategy and study selection process were published within the systematic review protocol [15].

### Data extraction

A standardized form was used to extract data from the studies for the quality assessment study and evidence synthesis. The detailed procedure was conducted as published in Domecky et al. 2021 [15].

### Quality assessment

The risk of bias assessment was conducted by two authors in duplicate (PD, ARP), using the Quality Assessment of Diagnostic Accuracy Studies (QUADAS-2) [16]. The Standards for Reporting of Diagnostic Accuracy Studies guideline 2015 (STARD) [17] was used to assess a list of essential items to ensure that the report of diagnostic accuracy study contains all the necessary information. The Cochrane Review Manager (RevMan, version 5.4.1; The Cohrane Collaboration, 2020) was used to extract the risk of bias assessment. Details of quality assessment are available in the published protocol [15].

### Data synthesis and analysis

Initially, this systematic review was conducted as a systematic review of diagnostic test accuracy studies. Despite the lack of data to calculate standardized effect sizes and high heterogeneity levels in the included studies, a narrative synthesis of findings was conducted. The detailed data synthesis and the analysis process were described in a published protocol [15].

### Statistical analysis

As for the baseline characteristics, there was no substantial clinical homogeneity observed in the studies regarding the participants and assessment methods. Therefore, meta-analysis was not conducted. For data extraction and quality assessment Microsoft Excel (Version 13,801.20864; Microsoft Inc, Redmond, WA, USA) was used. Furthermore, qualitative description was used to summarize the evidence.

### Subgroup analysis

The assessment suggested significant heterogeneity, therefore a subgroup analysis based on study-level characteristics was performed. This includes: diagnosis of the knee or hip osteoarthritis, type of surgery, use of antibiotic prophylaxis, average duration of follow-up, exact time of performed blood tests, and laboratory analysis.

## Results

### Study search and study characteristics

**Fig. 1:**
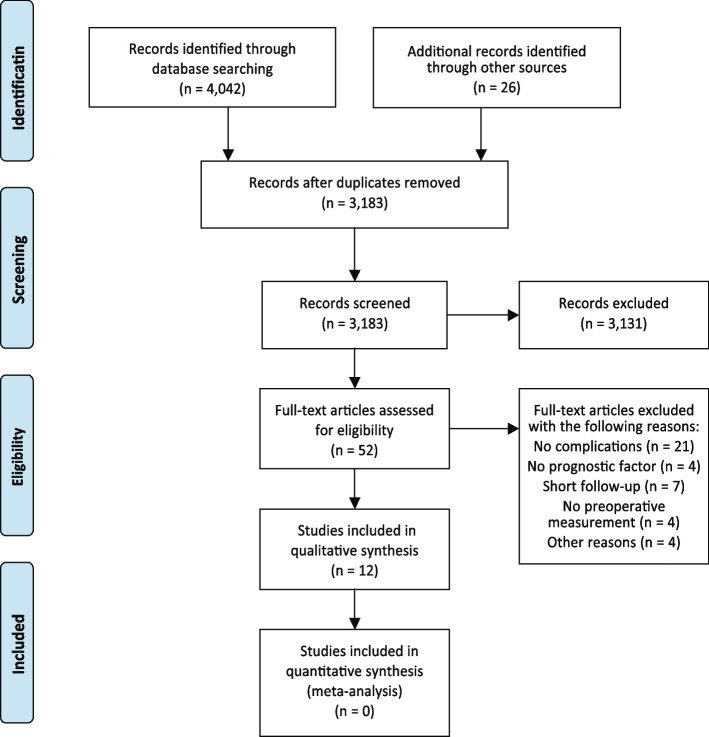
The PRISMA flow chart. This flow diagram demonstrates the search strategy and exclusion or inclusion criteria. Criteria included english language, year: from 1902 to January 31, 2020, primary arthroplasty, inflammatory blood parameters and preoperative and/or postoperative measure of these parameters, and minimum follow-up for of 30 days. All results that not included complications, prognostic factors (inflammatory blood parameters), short follow-up, no preoperative measurement were excluded. For more information see previously published protocol [15]

Systematic research identified 4,068 articles. The details of the selection are shown in the PRISMA flow chart (Fig. 1). Nevertheless, only 12 studies met the entry criteria. Baseline characteristics of the included studies are presented in Table 1.

**Table 1:** Baseline characteristics of the included studies

Study	Study design	Patients	Population	Exclusion criteria	Joint	Inflammatory parameter	Preoperative measure	Postoperative measure	Antibiotic prophylaxis	Follow-up
Carlsson et al. 1978 [18]	ND^−^	93	ND osteoarthritis	Patients with rheumatoid arthritis, pelvic spondylitis or with any other known cause of an elevated ESR	Hip	ESR	Yes (ND)	Yes (ND)	ND	49 months
Falzarano et al. 2017 [19]	Retrospective cohort study	1.248	ND osteoarthritis	Voluntary withdrawal from the scheduled follow-up program	Hip	CRP, ESR, PCT	Yes (1 h)	Yes (15 days, 1, 3, 6, 12, 24 and 36 months)	Yes	36 months
Forster et al. 1982 [20]	Retrospective comparative study	89	ND osteoarthritis	Patients with other forms of inflammatory arthritis	Knee	ESR	Yes (ND)	Yes, 48 h, 6 weeks, 3 months, 6 months, 1 year and yearly there after	ND	24 months
Mulier et al. 1973 [21]	Prospective cross-sectional	162	ND	ND	Hip	ESR, WBC	Yes (ND)	Yes (twice a week during postoperative stay (average time 30 days), and afterwards at monthly intervals)	Yes	20 months
Mumingjiang et al. 2014 [22]	Prospective cross-sectional	31	Primary osteoarthritis, traumatic osteoarthritis, rheumatoid athritis and osteochondritis dissecancs	Preoperative active inflammatory arthritis, complications in the affected knee during the follow-up period and history of knee infections	Knee	IL-6, CRP, ESR	Yes (ND)	Yes, day 1 and 7 and 1, 3 and 6 months (for complicated in the time of infection detection)	Yes	6 months
Okafor et al. 1998 [23]	Prospective cross-sectional	66	Degenerative arthritis or hip fractures	Preoperative sepsis, malignancy, RA, connective tissue disease or immunosuppressive treatments	Hip	ESR, CRP	Yes, on admission	Yes (day 2, 7 and 21)	Yes	12 weeks
Sanzén et al. 1997 [24]	Retrospective study	23	Arthrosis, failed osteosynthesis of femoral neck fracture, congenital dislocation of the hip, idiopathic femoral head necrosis	RA	Hip	ESR, CRP	Yes (ND)	Yes (6, (12) and last one depends on patient)	ND	7 weeks
Sastre et al. 2006 [25]	Prospective cross-sectional	143	Primary osteoarthritis	ND	Hip, knee	CRP	Yes (1 day)	Yes (2, 5 and 15 days)	Yes	2 years
Shah et al. 2009 [26]	Prospective case–control study	49	ND osteoarthritis	Known chronic inflammatory disease (RA, SLE, CD, Hashimoto's thyroiditis, psoriasis), recent antibiotic treatment or intercurrent infections before surgery, Paget's disease, revision arthroplasty, vascular disorders (lymphoproliferative disorders, autoimmune haemolytic anaemia) or cancer	Hip, knee	IL-6 (and other cytokines)	Yes (2 weeks)	Yes (6 h, 48 h, 6 weeks)	ND	6 weeks
Windisch et al. 2016 [27]	Retrospective study	1.068	Primary knee osteoarthritis	Active inflammation, active rheumatoid arthritis, lupus erythematosus, HIV, an inflammatory disease of chest or abdomen, surgical intervention in the 3 months prior to the planned TKA	Knee	CRP	Yes (ND)	Yes (from the first to the tenth day)	Yes	1 month
Wroblewski et al. 1974 [28]	ND^a^	100	Primary or secondary osteoarthritis	RA, suspected clinical or radiological sepsis, systemic disease likely to affect ESR, previous surgery	Hip	ESR	Yes (ND)	Yes (ND)	ND	4–7 years
Zarghooni et al. 2019 [29]	Prospective case–control study	41 (102)	ND	ND	Knee (or spine)	CRP, IL-6 (and other cytokines)	Yes (Control: 7 to 1 days, Case: 5 to 1 days)	Yes (Control: 0–22 days, Case: 0–197 days)	Yes	1 year after hospital discharge

*CD* Crohn's disease, *CRP* c-reactive protein, *ESR* Erythrocyte sedimentation rate, *HIV* Human immunodeficiency virus, *IL-6* Inteleukin-6, *ND* Not defined, *PCT* Procalcitonin, *RA* Rheumatoid arthritis, *SLE* Systemic lupus erythematosus, *TKA* Total knee arthroplasty, *WBC* White blood cell count

^a^Study design was not defined in methodology

### Inflammatory blood parameters

No eligible studies were found for NLR to estimate its prognostic value for IAI after primary THA or TKA. General information about the parameters is available in Table 2.

**Table 2:** General information about inflammatory blood parameters

**Inflammatory blood parameter**	**Number of studies**	**Patients**	**Gender**	**Hip patients**	**Knee patients**	**Age (years)** **(mean and SD)**	**Follow-up (months)** **(mean and SD)**	**Increase** **from PRV**^a^	**Decrease from POV**^a^
C-reactive protein	7	2,618	1,149 male 1,408 female 61 not specified	1,405	1,213	69.9 (SD 2,93)	16.2 (SD 13.68)	0–2 days	4–10 days
Interleukin-6	2	121	25 male 53 female 43 not specified	17	104	63.2 (SD 5.58)	6.5 (SD 5.27)	0–6 h	up to 48 h
Erythrocyte sedimentation rate	8	1,812	790 males 830 females 192 not specified	1,692	120	66.9 (SD 5.86)	23,6 (SD 19.19)	0–1 days	3–12 months

*PRV* Preoperative value, *POV* postoperative value, *SD* Standard deviation

^a^in case of non-complicated postoperative course

### C-reactive protein [19, 22–25, 27, 29]

One study found that CRP is more accurate than X-rays in predicting late chronic and early postoperative infections [22]. Although specific cut-off values for CRP have not been determined, other studies suggest that increased preoperative levels of 5 mg/l or higher can be a reliable predictor of implant-associated infection (IAI) and septic revision [23, 27]. In some cases, postoperative CRP levels can also predict the likelihood of complications. For example, one study found that postoperative CRP levels under 6 mg/l on the fifth day after surgery can predict an uncomplicated recovery. However, another study identified the seventh day as an indicator of infection [24]. Interestingly, CRP behaves similarly to interleukin-6 (IL-6) but differently from the erythrocyte sedimentation rate (ESR) [22]. Despite the potential benefits of using CRP to predict infections, some studies suggest that it may not be a reliable diagnostic marker for IAI [19, 29]. In fact, there's a risk of missed infections due to its relatively low sensitivity [25].

### Interleukin-6 [22, 26, 29]

Mumingjiang et al. 2014 [22] found that IL-6 had similar kinetics to CRP, with levels almost 7 times higher in patients with late IAI compared to those without infection. However, preoperative levels did not appear to be a reliable predictor of infection risk. In the study by Zarghooni et al. 2019 [29], patients with IAI had significantly higher levels of IL-6, particularly within the first 2 days postoperatively, with a 5.7-fold increase compared to controls. Unfortunately, only IL-1ra and IL-8 were found to be suitable as prognostic cytokines before surgery. Shah et al. 2009 [26] also found that IL-6 was a key marker for infection, with levels 3 times higher in patients with a proven deep infection compared to the control group. In contrast, levels of monocyte chemoattractant protein-1 (MCP-1) were lower in the infected group. The combination of increased IL-6 at 6 h and reduced MCP-1 at 48 h was associated with infection. These studies suggest that monitoring IL-6 levels may be a more effective method for predicting infections after joint replacement surgery compared to CRP. By understanding the relationship between cytokine levels and infection risk, healthcare providers can take appropriate measures to prevent further complications.

### Erythrocyte sedimentation rate [18–24, 28]

According to Sanzén et al. 1997 [24], ESR could be a more reliable marker than CRP in cases of chronic low-grade IAI, as some patients showed increased ESR with normal CRP values. Similarly, Carlsson et al. 1978 [18] found that ESR levels of 40 mm/hr or higher indicated deep infection more than three months postoperatively, even in the absence of symptoms or radiographic signs of infection. Mulier et al. 1973 [21] observed that hip replacements were associated with long-term elevated ESRs, which eventually normalized within the first four months after operation. Forster et al. 1982 [20] compared ESR levels in uncomplicated THA with those in IAI and found significantly higher ESR levels in patients with IAI. In case of uncomplicated surgery, ESRs fell to 20 mm/hr within six months and the higher rate after surgery did not imply infection, especially when participants presented a high preoperative level. In the study by Wroblewski et al. 1974 [28], the ESR varied from 30 mm/hr to 140 mm/hr in cases of sepsis after arthroplasty. Preoperative high ESR levels in this study did not predict IAI. Falzarano et al. 2017 [19] concluded that there are two circumstances when the ESR can be a better IBP than CRP or PCT: first, some low-grade bone infections and second, joint infections, both due to the low-level pathogens. In the study by Okafor et al. 1998 [23], there was a significant difference in ESR levels between the normal and the infected group from the second postoperative day (71 vs 91,6 mm/hr) until day 21 (36.5 vs 71.2 mm/hr). The authors thus suggested repeated blood tests between day 7 and 21 after operation to detect early infection. Mumingjiang et al. 2014 [22] observed that ESRs increased and peaked 7 days after surgery and returned to baseline levels within 3 months. There was a significant difference when comparing the uninfected group’s ESR levels to the infected group’s ESR levels at six months following surgery (10.6 vs 42.6 mm/hr).

### White blood cell count and procalcitonin [19, 21]

Both WBC and PCT were separately analyzed in only one of the included studies. Therefore, there is no additional benefit in the narrative synthesis for these IBP.

### Risk of bias

**Fig. 2:**
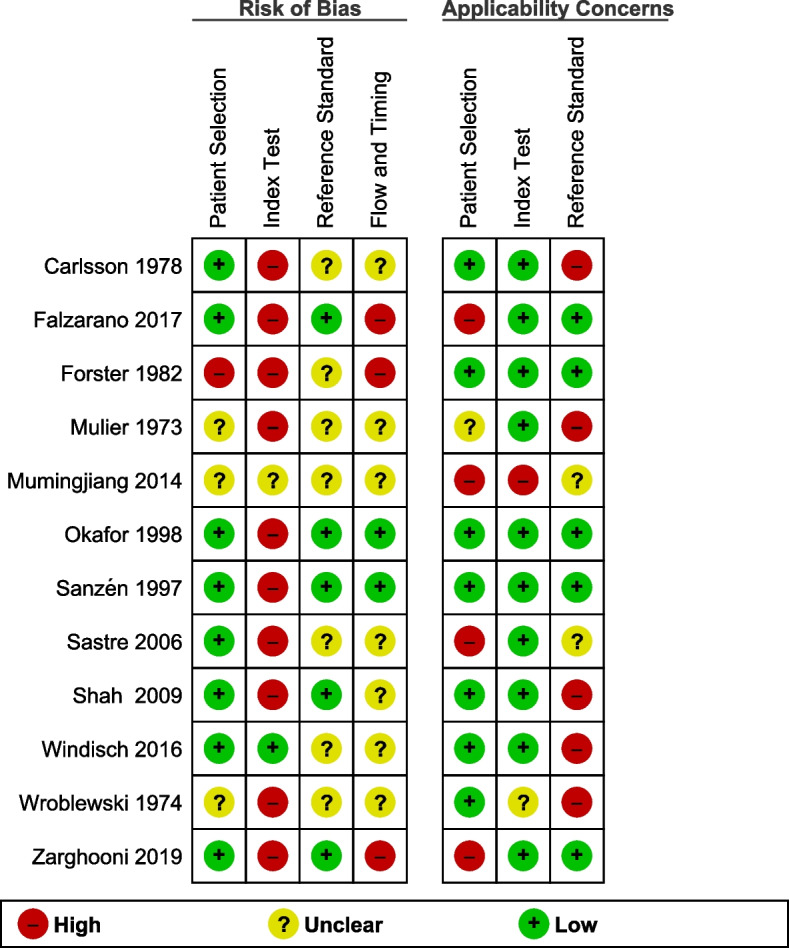
Risk of bias and applicability concerns summary (QUADAS-2). This figure demonstrates the risk of bias and applicability concerns summary (QUADAS-2). The overall extracted data suggested that not all the included studies were conducted according to the STARD 2015 guidelines [16, 17]

The risk of bias assessment is shown in Fig. 2. The overall extracted data suggested that not all the included studies were conducted according to the STARD 2015 guidelines [17]. Scores of each included study are shown in Table 3.

**Table 3:** Standards for reporting diagnostic accuracy checklist

**Study**	**Carlsson et al. 1978** [18]	**Falzarano et al. 2017** [19]	**Forster et al. 1982** [20]	**Mulier et al. 1973** [21]	**Mumingjiang et al. 2014** [22]	**Okafor et al. 1998** [23]	**Sanzén et al. 1997** [24]	**Sastre et al. 2006** [25]	**Shah et al. 2009** [26]	**Windisch et al. 2016** [27]	**Wroblewski et al. 1974** [28]	**Zarghooni et al. 2019** [29]
Title or abstract	0/1	0/1	0/1	0/1	0/1	0/1	0/1	1/1	0/1	1/1	0/1	0/1
Abstract	0/1	0/1	0/1	0/1	0/1	0/1	0/1	0/1	0/1	0/1	0/1	0/1
Introduction	2/2	2/2	2/2	1/2	2/2	2/2	2/2	2/2	2/2	2/2	2/2	2/2
Methods	6/17	6/17	8/17	1/17	8/17	8/17	8/17	4/17	9/17	9/17	3/17	8/17
Results	0/8	3/8	0/8	0/8	1/8	1/8	1/8	4/8	2/8	2/8	1/8	3/8
Discussion	1/2	1/2	1/2	0/2	1/2	1/2	1/2	1/2	2/2	2/2	1/2	2/2
Other information	1/3	1/3	0/3	0/3	0/3	0/3	0/3	0/3	0/3	1/3	0/3	1/3
Overall score	10/30	13/30	11/30	2/30	12/30	12/30	12/30	12/30	15/30	17/30	7/30	16/30

## Discussion

To our knowledge, this is the first systematic review focused on IBP response to trauma induced by orthopedic surgery and evaluation of potential clinical usefulness of IBP as predictive factors for implant-associated infections in patients who are at higher risk of developing IAI before undergoing surgery. All the literature eligible according to the inclusion and exclusion criteria was included, thus obtaining 12 studies in total. After data extraction and summarizing all the evidence, no quantitative analysis – including the diagnostic accuracy statistics – could be performed. The included studies were inadequate as they lacked data related to QUADAS-2, or not respecting STARD criteria [16, 17]. Moreover, there were neither enough data nor sufficient non-conflicting evidence to support any prognostic potential of the analyzed IBPs for patient risk stratification and to suggest its routine use to predict IAI in relation to preoperative and postoperative levels of the analyzed IBP. At the same time, no eligible studies were found for NLR after primary THA or TKA.

After a total joint arthroplasty, IL-6 serum levels may increase to 30–430 pg/mL for up to three days before returning to normal levels, if the surgery is uncomplicated [10]. Zarghooni et al. 2019 [29] studied other cytokines and MCPs to determine their potential as prognostic factors in addition to IL-6. They found that IL-1ra and IL-8 showed the most convincing evidence, but IL-6 was confirmed only for diagnostic purposes. Combining IL-6 with MCP-1 may show a specific trend in predicting IAI, but its pharmacokinetics varies depending on tissue damage [10, 22, 26, 29].

The hepatocytes produce CRP, an acute-phase protein, in response to inflammation, infection, and neoplasm. While CRP is not 100% sensitive and low-grade or encapsulated infections may result in less intensive systemic reactions, healthy patients without inflammation, infection, or neoplasm typically exhibit low serum concentrations of CRP. Following surgery, CRP levels peak within two to three days and return to normal approximately three to eight weeks after surgery, making it a recommended marker for monitoring the postoperative course. Unfortunately, there is significant inter-individual variation in CRP levels during the first week after surgery across studies, although subsequent decreases follow a similar pattern [30–33]. According to the included studies, CRP levels can predict an uncomplicated postoperative course. The potential prediction for early or later infection remains unknown. Therefore, it is not appropriate to predict postoperative complications based on this parameter [19, 23, 24, 27].

The relative lack of specificity limits the ESR usage especially in patients suffering from undergoing inflammatory joint disease such as rheumatoid arthritis [22]. However, the ESR may potentially be used in cases where the normal assay of CRP was found, particularly in some chronic low-grade IAI [24]. Also, in cases of delayed infection since the increased levels due to its kinetics remain high for approximately three to twelve months [18]. An exciting feature of the ESR is that even if it remains elevated for a long time after surgery because of infection, the ESR quickly drops to preoperative levels after the infection is healed [18–24, 28].

PCT detection is useful for patients with sepsis due to its properties. However, it can also increase in other inflammatory conditions such as major surgery, including primary THA and TKA. After surgery, PCT levels peak on the first day and quickly return to pre-intervention levels within 6 to 14 days. Unfortunately, there is insufficient precise information about PCT induction in relation to postoperative conditions. Typically, the increase in PCT concentration within the first day after surgery does not surpass levels associated with bacterial infections [30, 34–36].

A sensitivity of 45% limits the usage of WBC. Although the higher specificity of 87% may be helpful in some specific situations, peak levels are usually observed within 2–3 days after surgery [37].

The diagnostic criteria of IAI have been changing over time as knowledge and evidence have progressed. In 2013, the Infection Diseases Society of America (IDSA) modified the definition [38]. In addition to MSIS and IDSA criteria, the International Consensus Meeting (ICM) in 2013 adapted a definition from Parvizi et al. 2013 [39, 40]. The MSIS, IDSA and ICM criteria are widely used for IAI diagnosis [38, 40]. Unfortunately, an accurate determination can still not be made using the current diagnostic criteria (especially the ICM and MSIS criteria) [41]. Exactly for that reason a new definition has been proposed establishing an evidence-based and weight-adjusted scoring system in 2018 [42].

The evidence-based stepwise algorithm for an IAI diagnosis is established according to major criteria (i.e., the microbiological confirmation of positive cultures, a sinus tract with evidence of communication to the joint, or visualization of the prosthesis), as well as to minor criteria (synovial inflammatory parameters or IBP), also based on intraoperative diagnoses. Each of the criteria is individually scored to reach the final decision [42]. Since the studies included in our study were conducted in different periods of time, all the facts discussed above resulted in discrepancies among the reference standards. Therefore, various criteria for reference standards as’IAI diagnoses’ were used in the included studies.

The strength of the present study stems from a thorough search in all of the essential databases and the review of the relevant literature; at least two independent reviewers collecting data, including a third reviewer to judge disagreements and assess the risk of bias; the quality of the included studies according to the QUADAS-2, the STARD Guideline 2015; and finally, the appropriate set of eligibility criteria [16, 17].

Nevertheless, the present study has several limitations. Except for the study done by Windisch et al. 2016 [27], data were limited because the included studies were found lacking in diagnostic accuracy statistics, including specificity, sensitivity, and area under the receiver operating characteristics curve. Therefore, no quantitative synthesis, or its associated components, were performed. On the other hand, due to the prospective nature of most of these studies, there was a fair amount of withdrawal. Preoperative and postoperative measurements were differently applied across the included studies, resulting in a potential selection bias and a possible exaggeration of the outcome. Notably, no strict case definition for IAI was used through the studies as the reference standard as’IAI diagnoses’ evolved over the time. This could have led to a classification bias between the infected and non-infected patients regarding each study reference standards. Finally, variation was observed among cut-off values of the index tests used in the included studies.

## Conclusion

This systematic review showed that there are insufficient data available to support any prognostic potential of the analyzed IBP for patient risk stratification based on these IBP only. CRP had better diagnostic accuracy than X-rays in predicting late chronic and early postoperative infections. However, no specific cut-off values were detected. IL-6 was found to be a suitable marker for predicting IAI, while its kinetics were found to be the same as CRP kinetics in an uncomplicated postoperative course. ESR was found to be a better indicator for some low-grade bone infections and joint infections. Nevertheless, there is also a potential for other cytokines (IL-1ra, IL-8) or MCP-1 for preoperative and postoperative routine screening. Still, the heterogeneity of the included studies suggested a need for further research to establish reliable cutoff values for the IBPs.

## Data Availability

All data generated or analysed during this study are included in this published article.
